# High Molecular Weight Hyaluronan Suppresses Macrophage M1 Polarization and Enhances IL-10 Production in PM_2.5_-Induced Lung Inflammation

**DOI:** 10.3390/molecules24091766

**Published:** 2019-05-07

**Authors:** Qiwen Shi, Lan Zhao, Chenming Xu, Leifang Zhang, Hang Zhao

**Affiliations:** 1 Collaborative Innovation Center of Yangtze River Delta Region Green Pharmaceuticals, Zhejiang University of Technology, Hangzhou 310014, China; 17326058655@163.com (L.Z.); dg1830070@smail.nju.edu.cn (C.X.); zhaohang@zjut.edu.cn (H.Z.); 2 Zhejiang Provincial Engineering Technology Research Center of Marine Biomedical Products, School of Food and Pharmacy, Zhejiang Ocean University, Zhoushan 316022, China; 1111423109@zjut.edu.cn

**Keywords:** hyalruonan, macrophage polarization, PM_2.5_, inflammation, IL-10, cytokines

## Abstract

PM_2.5_ is particulate matter with a diameter of 2.5 μm or less. Airway macrophages are the key players regulating PM_2.5_-induced inflammation. High molecular weight hyaluronan (HMW-HA) has previously been shown to exert protective effects on PM_2.5_-induced acute lung injury and inflammation. However, little is known about the detailed mechanism. In this study, we aimed to determine whether HMW-HA alleviates PM_2.5_-induced pulmonary inflammation by modulating macrophage polarization. The levels of M1 biomarkers TNF-α, IL-1β, IL-6, CXCL1, CXCL2, NOS2 and CD86, as well as M2 biomarkers IL-10, MRC1, and Arg-1 produced by macrophages were measured by ELISA, qPCR, and flow cytometry. In addition, the amount of M1 macrophages in lung tissues was examined by immunofluorescence of CD68 and NOS2. We observed a decline in PM_2.5_-induced M1 polarization both in macrophages and lung tissues when HMW-HA was administered simultaneously. Meanwhile, western blot analysis revealed that PM_2.5_-induced JNK and p38 phosphorylation was suppressed by HMW-HA. Furthermore, in vitro and in vivo studies showed that co-stimulation with HMW-HA and PM_2.5_ promoted the expression and release of IL-10, but exhibited limited effects on the transcription of MRC1 and ARG1. In conclusion, our results demonstrated that HMW-HA ameliorates PM_2.5_-induced lung inflammation by repressing M1 polarization through JNK and p38 pathways and promoting the production of pro-resolving cytokine IL-10.

## 1. Introduction

Fine particulate matter with an aerodynamic diameter less than 2.5 μm (PM_2.5_) is a serious air pollutant associated with adverse health effects including airway damage, cardiovascular impairment, metabolic disruption, and immunity disorders [1]. Real-time PM_2.5_ air quality index (AQI) is monitored by the China Meteorological Bureau as an indicator of daily air pollution level. Chronic exposure to PM_2.5_ has been documented to trigger persistent pro-inflammatory responses in the respiratory system, such as elevated production of reactive oxygen species (ROS) and pro-inflammatory cytokines, neutrophil infiltration, accumulation of intracellular Ca^2+^, and altered macrophage polarization, resulting in declined lung function, exacerbation of bronchitis, asthma, chronic obstructive pulmonary disease (COPD) and pulmonary fibrosis, and increased morbidity and mortality of lung cancer [2,3,4]. Huge efforts have been made by the Chinese government to limit the production of PM_2.5_, such as reducing emission from cars and coal-fired power plants, but it is impossible to resolve this environmental issue in a short-term. Seeking effective agents to against PM_2.5_-related respiratory damage is a practical strategy to protect human health. 

Alveolar macrophages (AMs) are the primary cells processing environmental micro-particles in the lungs and are the first line of defense against noxious particles. Macrophages secrete inflammatory cytokines upon PM_2.5_ exposure, and are also required for PM_2.5_-stimulated T cell cytokine secretion and granzyme production, leading to the long-term pro-inflammatory status of T cells and the death of epithelial cells [3,5]. Theoretically, macrophage polarization has two extremes, classically activated state (M1) and alternatively activated state (M2), which are associated with T-helper cell polarization [6]. M1 macrophages are polarized in response to microbial products or pro-inflammatory cytokines including lipopolysaccharide (LPS), tumor necrosis factor-α (TNF-α), and interferon-γ (IFN-γ), thereby releasing inflammatory mediators that facilitate the clearance of infecting pathogens or harmful matters [7]. On the contrary, M2 macrophages are stimulated by various factors—such as macrophage colony stimulating factor (M-CSF), interleukin 4 (IL-4), IL-13, and transforming growth factor-β (TGF-β)—and participate in the resolution of inflammation and tissue repair partially through the production of anti-inflammatory factors [8]. It is noteworthy that M1-M2 polarization is a tightly controlled dynamic process. An imbalance in M1-M2 polarization is often observed under pathological conditions such as asthma, obesity, and cancer, and promotes disease progression [9]. Therefore, targeting molecules that regulate macrophage polarization or reprogramming is an attractive strategy to treat diseases [10]. 

PM_2.5_ elevates ROS level in macrophages and meanwhile is recognized by Toll-like receptors 4 (TLR4) and TLR2 to induce or exacerbate acute inflammation and activate M1 polarization [5,11,12]. JNK and p38 are the downstream effectors of TLRs activation and oxidative stress, and are involved in inflammatory responses and M1 phenotype activation [13,14]. Therefore, it is possible that JNK and p38 are participated in PM_2.5_-induced macrophage polarization. Besides TLRs, other receptors such as IFN-γR and nucleotide-binding oligomerization domain (NOD)-like receptors (NLRs) also participate in M1 polarization [6]. The pathways involved in M2 polarization include signal transducers and activators of transcription 3 (STAT3), STAT6, and interferon regulatory factor 4 (IRF4) [6].

Hyaluronan (HA) is a linear and unbranched glycosaminoglycan of extracellular matrix (ECM), and is composed of repeating disaccharide units of d-glucuronic acid and *N*-acetylglucosamine. By interacting with a variety of surface receptors and intracellular proteins, HA displays important functions in a number of biological and pathological processes such as development, T cell recruitment and activation, tissue injury and repair, and cancer metastasis [15]. Depending on the molecular weight, HA can be classified into four groups: high molecular weight HA (HMW-HA, >1000 kDa), medium molecular weight HA (MMW-HA, 250–1000 kDa), low molecular weight HA (LMW-HA, 10–250 kDa), and HA oligomers (<10 kDa) [16]. The molecular weight influences the binding proteins, biological responses, and therapeutic uses of HA. Generally, smaller HA fragments are formed during pathological statuses such as inflammation, oxidative stress, fibrosis, and cancer, exhibiting pro-inflammatory potentials. In the opposite, HMW-HA is the native state existing under homeostasis, eliciting protective actions against inflammation [17]. The therapeutic effects of HWM-HA have been validated in multiwalled carbon nanotubes (MWCNT)-induced lung injury, LPS-increased pulmonary vascular permeability, and meconium-impaired lung function [18,19,20]. 

CD44 is the major cell surface hyaluronan receptor highly expressed by AMs. The CD44 expressed by AMs constitutively binds HA, while other macrophages such as peritoneal macrophages and M-CSF-derived macrophages are unable to bind HA [21,22]. CD44-deficient AMs fail to bind HA and are more susceptible to apoptosis, indicating a role of CD44-HA binding in AM survival. Moreover, the interaction of CD44 and HMW-HA has been reported to contribute to anti-inflammatory effects [23]. CD44^-/-^ mice exhibit impaired efficiency and effectiveness in the resolution of inflammation by showing persistence accumulation of LMW-HA and impaired clearance of apoptotic neutrophils.

Our previous work demonstrated that HMW-HA treatment alleviated PM_2.5_-induced acute lung injury and inflammation. Rats co-treated with PM_2.5_ and HMW-HA showed reduced numbers of total cells and neutrophils and lower levels of TNF-α and IL-1β in bronchoalveolar lavage fluid (BALF) compared with rats exposed to PM_2.5_ alone [24]. In this study, we aimed to investigate the effect of PM_2.5_ and HMW-HA on macrophages. PM_2.5_ has been shown to stimulate macrophages to produce pro-inflammatory cytokines and disrupt M1-M2 balance, and meanwhile, HMW-HA exhibits a tendency to drive macrophage polarize toward M2 phenotype and away from M1 phenotype [5,25]. Therefore, it was hypothesized that HMW-HA might attenuate PM_2.5_-induceed lung inflammation by modulating macrophage polarization. The expression of major M1 and M2 biomarkers was tested in this work, and the results revealed that HMW-HA reduced PM_2.5_-induced inflammation and M1 polarization at least in part through inhibiting JNK and p38 activation, and elevated IL-10 secretion rather than M2 activation to resolve inflammation. 

## 2. Results

### 2.1. Assessment of Macrophage Phenotype in Response to PM_2.5_

**Figure 1:**
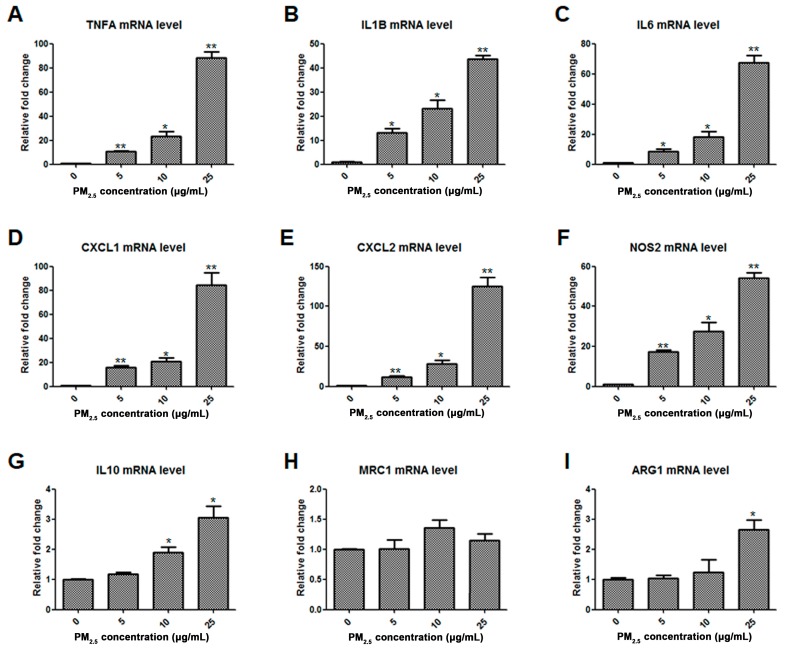
Effects of PM_2.5_ on macrophage polarization. NR8383 cells were treated with 0, 5, 10, and 25 μg/mL PM_2.5_ for 6 h, and the mRNA levels of TNFA (**A**), IL1B (**B**), IL6 (**C**), CXCL1 (**D**), CXCL2 (**E**), NOS (**F**), IL10 (**G**), MRC1 (**H**), and ARG1 (**I**) were measured by real-time RT-PCR. Data are presented as mean ± SD, and represent three independent experiments. * *p* < 0.05 and ** *p* < 0.01, compared with the control group treated without PM_2.5_.

To investigate the impact of PM_2.5_ on resting macrophages, NR8383 rat AMs were exposed to 0, 5, 10, and 25 μg/mL of PM_2.5_ for 6 h, and the mRNA levels of M1- or M2-associated cytokines, chemokines and key molecular markers were determined by quantitative RT-PCR. The transcription of TNFA, IL1B, IL6, chemokine C-X-C-motif ligand 1 (CXCL1), CXCL2, and nitric oxide synthase 2 (NOS2) was significantly upregulated by PM_2.5_ in a concentration-dependent manner (Figure 1A–F), while the expression of M2 phenotype markers IL10, MRC1, and ARG1 was less affected with a 3-fold induction at most (Figure 1G–I). These data suggested that PM_2.5_ exposure stimulated resting macrophages mostly toward the classically activated state, not the alternatively activated state. 

### 2.2. HMW-HA Attenuated PM_2.5_-Induced Production of Pro-Inflammatory Mediators

**Figure 2:**
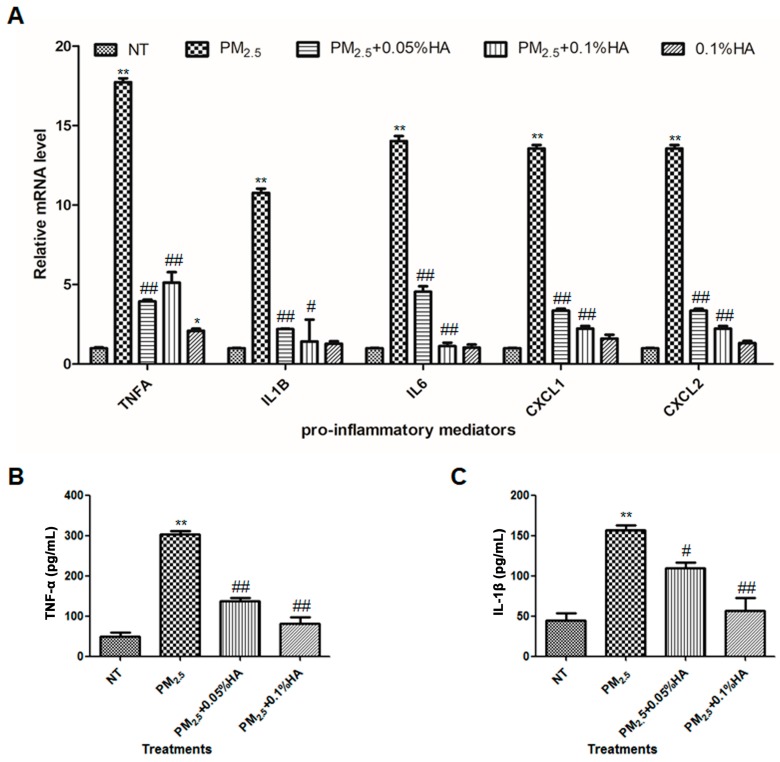
Anti-inflammatory effects of HMW-HA on PM_2.5_-treated macrophages. (**A**) NR8383 cells were exposed to PBS, PM_2.5_, 0.1% HMW-HA, PM_2.5_ and 0.05% HMW-HA, or PM_2.5_ and 0.1% HMW-HA simultaneously for 6 h, and the mRNA expression of TNFA, IL1B, IL6, CXCL1, and CXCL2 was determined by real-time RT-PCR. (**B**,**C**) The secretion of TNF-α and IL-1β by NR8383 cells 24 h after indicated treatments was assessed by ELISA. Data are presented as mean ± SD, and represent three independent experiments. * *p* < 0.05 and ** *p* < 0.01, compared with the no treatment (NT) group. # *p* < 0.05 and ## *p* < 0.01, compared with cells exposed to PM_2.5_ alone.

Previously, we reported that HMW-HA ameliorated PM_2.5_-induced acute lung injury by suppressing epithelial apoptosis [24]. Here, we examined whether HMW-HA could repress the expression of inflammatory cytokines and chemokines induced by PM_2.5_. Based on the dose–response results, we chose 10 μg/mL as the concentration of PM_2.5_ for further study, which is sufficient to trigger inflammatory responses. As shown in Figure 2A, HMW-HA successfully reduced the mRNA levels of TNFA, IL1B, IL6, CXCL1, and CXCL2 upregulated in response to PM_2.5_. HMW-HA alone slightly increased the transcription of TNFA by approximately 2 folds, and exhibited no effect on the expression of other pro-inflammatory mediators (Figure 2A). Consistent with the results from real-time RT-PCR, PM_2.5_-induced TNF-α and IL-1β secretion was decreased by HMW-HA (Figure 2B,C). Thus, it is confirmed that HMW-HA suppressed macrophage inflammatory responses initiated by PM_2.5_.

### 2.3. HMW-HA Negatively Regulated PM_2.5_-Induced M1 Polarization

**Figure 3:**
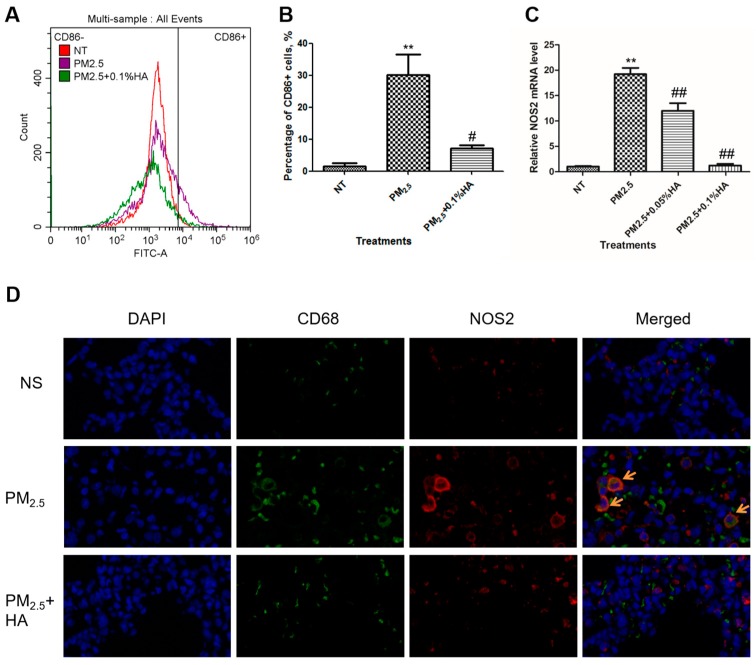
Inhibitory effects of HMW-HA on PM_2.5_-induced M1 polarization. (**A**) NR8383 cells were administered with PBS, PM_2.5_, or PM_2.5_ and HMW-HA simultaneously for 24 h, and CD86 (M1 marker) protein expression level was determined by flow cytometry. (**B**) The percentage of CD86-positive cells was calculated. Data were presented as mean ± SEM of three independent experiments. (**C**) The mRNA level of NOS2 (M1 marker) was determined by real-time RT-PCR after NR8383 cells were treated as indicated for 6 h. Data are presented as mean ± SD, and represent three independent experiments. ** *p* < 0.01, compared with the NT group. # *p* < 0.05 and ## *p* < 0.01, compared with cells exposed to PM_2.5_ alone. (**D**) Rats were exposed to NS, PM_2.5_ or PM_2.5_ + 0.2% HA for three consecutive days by intratracheal instillation. Lung tissues were counterstained with anti-CD68 (macrophage marker, green) and anti-NOS2 (M1 marker, red) antibodies, and nuclei were stained with DAPI (blue). Fluorescence was observed by fluorescence microscopy. Arrows indicate M1 macrophages.

CD86 is a surface marker for M1 macrophages [26]. We labeled macrophages by CD86-FITC antibody, and determined the fluorescence intensity of each macrophage by flow cytometry assay. The percentage of CD86-positive macrophages was elevated from 2% to 30% upon PM_2.5_ exposure, and dropped to 7% when co-treated with HMW-HA and PM_2.5_ (Figure 3A,B). The mRNA transcription of NOS2, another M1 marker, was also measured in order to assess the extent of M1 macrophage polarization [27]. Similarly, HMW-HA reduced NOS2 transcription provoked by PM_2.5_ (Figure 3C). The amount of M1 macrophages in rat lung tissues was detected by immunofluorescence staining of both CD68 (red) and NOS2 (green) protein. CD68 is the marker for macrophages [28]. The lung tissues from PM_2.5_-exposed rats contained a number of NOS2-positive macrophages, and HMW-HA treatment limited PM_2.5_-induced M1 polarization (Figure 3D). Both in vitro and in vivo results demonstrated that HMW-HA repressed M1 polarization caused by PM_2.5_.

### 2.4. HMW-HA Inhibited PM_2.5_-Induced Phosphorylation of JNK and p38

**Figure 4:**
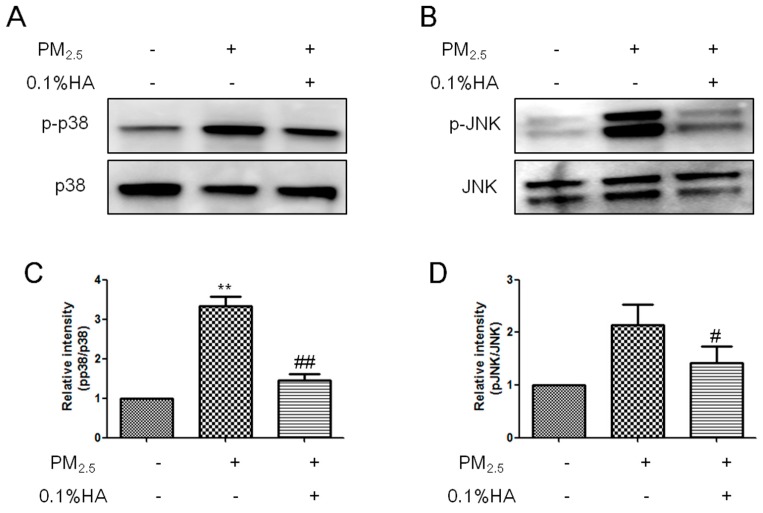
Effects of HMW-HA on PM_2.5_-induced JNK and p38 phosphorylation in NR8383 cells. (**A**,**B**) Cells were treated with PBS, PM_2.5_, or PM_2.5_ and HMW-HA simultaneously for 2 h, and the protein expression of p38, p-p38, JNK, and p-JNK was determined by western blot analysis. (**C**,**D**) Quantitative analysis of the blots is normalized to p38 and JNK, respectively. Each bar represents mean ± SEM of three independent experiments. ** *p* < 0.01, compared with the group treated without PM_2.5_ or HMW-HA. # *p* < 0.05 and ## *p* < 0.01, compared with cells exposed to PM_2.5_ alone.

JNK and p38 phosphorylation is the downstream of TLRs, directing resting macrophages toward M1 phenotype to release pro-inflammatory mediators [13]. Blockage of JNK or p38 pathway decreases the expression of M1 biomarkers, and activation of JNK and p38 promotes M2 macrophages to repolarize to M1 [29]. Here, we examined the levels of phosphorylated JNK and p38 protein after NR8383 were treated with PBS, PM_2.5_ or HA + PM_2.5_ for 2 h. Western blot analysis showed that PM_2.5_ enhanced JNK and p38 phosphorylation and co-administration with HMW-HA restrained PM_2.5_-induced JNK and p38 activation (Figure 4A–D). Our results indicated that HMW-HA blocked PM_2.5_-induced classical activation of macrophages at least partially through JNK and p38 pathways.

### 2.5. HMW-HA Stimulated IL-10 Production in the Presence of PM_2.5_

**Figure 5:**
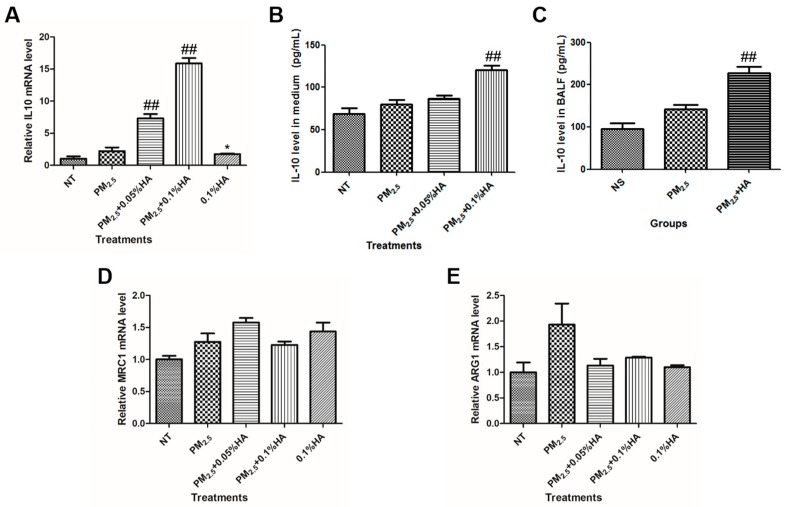
Upregulation of IL-10 in response to co-stimulation of PM_2.5_ and HMW-HA. (**A**) NR8383 cells were stimulated by PBS, PM_2.5_, 0.1%HMW-HA, PM_2.5_ and 0.05% HMW-HA, or PM_2.5_ and 0.1% HMW-HA simultaneously for 6 h, and IL10 mRNA level was determined. (**B**) The secretion of IL-10 by NR8383 cells 24 h after indicated treatments was assessed by ELISA. (**C**) Rats were exposed to NS, PM_2.5_ or PM_2.5_ + 0.2% HA for three consecutive days by intratracheal instillation, and the concentration of IL-10 in BALF was measured by ELISA. (**D**,**E**) The mRNA levels of MRC1 and ARG1 in NR8383 cells were tested after indicated treatments. Data from in vitro study were presented as mean ± SD, and represent three independent experiments. Data from in vivo study were presented as mean ± SEM (N = 6). * *p* < 0.05, compared with the NT group or the NS group. ## *p* < 0.01, cells or rats exposed to PM_2.5_ alone.

As previously reported, HMW-HA modulates the expression of biomarkers associated with alternatively activated state of macrophages [25]. Thus, we tested whether stimulation with PM_2.5_ and HMW-HA simultaneously would affect the expression of IL10, MRC1, and ARG1. The detection of mRNA level and protein secretion indicated that macrophages co-exposed to PM_2.5_ and HMW-HA expressed high levels of IL10 and HMW-HA alone were also able to induce IL10 transcription by 1.7 folds (Figure 5A,B). Moreover, IL-10 level in BALF from rats co-administered with PM_2.5_ and HMW-HA was approximately 2-fold higher than that from rats treated with PM_2.5_ alone (Figure 5C). Neither MRC1 nor ARG1 mRNA level was significantly changed in response to PM_2.5_, HMW-HA or co-treatment of PM_2.5_ and HMW-HA (Figure 5D,E). Together, our data illustrated that HMW-HA upregulated pro-resolving cytokine IL-10, but not M2 markers MRC1 and Arg-1.

## 3. Discussion

**Figure 6:**
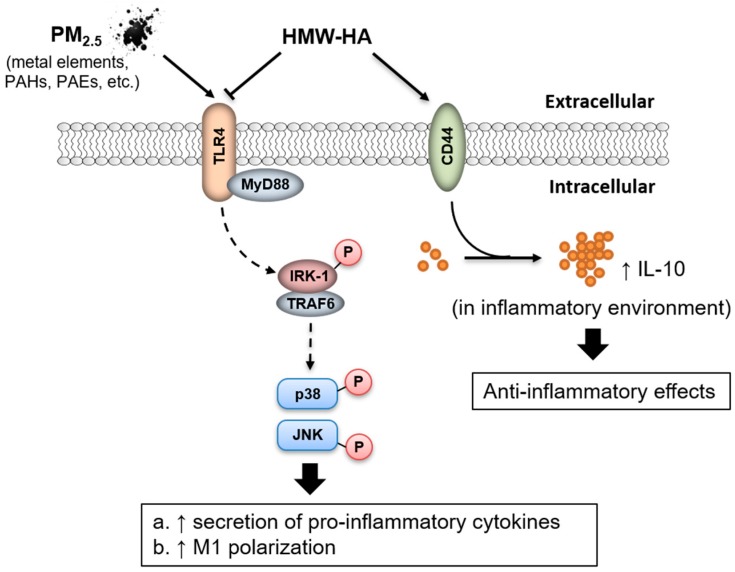
Schematic model for the effect of HMW-HA on PM_2.5_-mediated inflammation. HMW-HA represses PM_2.5_-induced M1 polarization and pro-inflammatory cytokine production, and co-treatment of PM_2.5_ and HMW-HA increases the level of pro-resolving cytokine IL-10.

In the present study, we reported that macrophages simultaneously stimulated by HMW-HA and PM_2.5_ exhibited reduced expression of pro-inflammatory mediators and M1 biomarkers, limited phosphorylation of JNK and p38 and elevated production of IL-10 compared to macrophages treated with PM_2.5_ alone (Figure 6). Immunofluorescence staining of rat lung tissues with CD68 and NOS2 antibodies confirmed the negative regulation of HMW-HA on PM_2.5_-induced M1 polarization, and IL-10 concentration in BALF supported the involvement of IL-10 in HMW-HA-mediated anti-inflammatory effects (Figure 6).

Our study found that PM_2.5_ mainly transformed resting macrophages to M1 phenotype that secretes an array of inflammatory cytokines and chemokines, with minor impact on the expression of M2 biomarkers IL-10, MRC1, or Arg-1. Zhao et al. have shown that PM_2.5_ stimulates macrophages to release pro-inflammatory mediators [5]. Furthermore, previous reports have revealed that PM_2.5_ is able to display synergistic effects with LPS via ROS production and MyD88 pathway [13,29]. However, the function of PM_2.5_ in M2 polarization depends on the stimulus or the pathological condition. PM_2.5_ counteracts with IL-4-induced M2 polarization partially through mammalian target of rapamycin (mTOR) pathway, whereas in ovalbumin (OVA)-induced asthma, PM_2.5_ aggravates allergic inflammation by activating M2 macrophages and subsequent Th2-related immune responses [21,30]. In addition, co-exposure to PM_2.5_ and OVA notably promotes the levels of M2 marker YM-1, IL-4, and IL-13 in murine macrophages [31]. In apoE-deficient mice, the upregulation of TNF-α, IL-6, NOS2, IL-12, MRC1, and Arg-1 has been observed in PM_2.5_-exposed group, implying an activation of both M1 and M2 macrophages [32]. The difference of macrophages in response to PM_2.5_ also probably attributes to the characteristics of macrophages, and our observation may be specific to AMs. One limitation of our study is that we only examined the resident AMs in lung tissues. We may further differentiate the AMs in BALF and investigate their polarization state. 

Our previous study has defined the major chemical components of PM_2.5_, including heavy metals, polycyclic aromatic hydrocarbons (PAHs), and phthalates. Different heavy metals may affect macrophages differently. For instance, lead induces the secretion of pro-inflammatory cytokines, while cadmium triggers the production of IL-4 and IL-10 [33]. Benzo[α]pyrene (BaP) is a type of HMW PAHs with a tendency to elicit genotoxic effects. The exposure to BaP results in impaired secretion of IL-1β, IL-6 and CXCL1, as well as enhanced release of TNF-α and IL-10 [34]. Diethyl and dibutyl phthalates increase the secretion of IL-6, IL-10, and CXCL8 by macrophages, but not TNF-α production [35].

In this study, we demonstrated that PM_2.5_ exposure caused JNK and p38 phosphorylation in AMs, and HMW-HA inhibited PM_2.5_-induced JNK and p38 activation to reduce M1 polarization and exhibit anti-inflammatory effects. We previously found that PM_2.5_ triggered BEAS-2B human bronchial epithelial cell apoptosis through ROS generation and subsequent activation of JNK and p38 signaling pathway, and HMW-HA acted as an antioxidant to protect PM_2.5_-induced lung injury [36]. ROS elevation has been observed in PM_2.5_-treated macrophages and is able to activate MAPK signaling to interfere with macrophage polarization [36,37]. Excessive ROS is associated with the phagocytic function of M1 macrophages and enhanced expression of pro-inflammatory genes, and ROS reduction is accompanied by reprogramming of M1 to M2 phenotype and decreased production of inflammatory mediators [13]. JNK and p38 phosphorylation leads to activation of transcription factors such as activating transcription factor (ATF) family, STAT family and AP-1 family, and subsequent expression of inflammatory genes [38,39].

The present study showed that co-exposure to HMW-HA and PM_2.5_ significantly increased IL-10 expression and secretion in both NR8383 cells and rat models. IL-10 is an immunosuppressive cytokine with the ability to resolve inflammation and promote wound repair. The upstream regulators of IL-10 include TLRs, extracellular signal-regulated kinases (ERKs), NOD2 and type I IFNs signaling, and the exact molecular mechanism regulating IL-10 production may vary depending on the type of immune cells [40]. After secreted, IL-10 binds to IL-10 receptor (IL-10R) to modulate signaling pathways such as Janus kinase (JAK)/STAT and Akt, subsequently decreasing the production of pro-inflammatory mediators and initiating the expression of anti-inflammatory genes and miRNAs [37,41]. Similar to our finding, Gebe et al. indicated that IL-10 is indispensable in thiolated HMW-HA-prevented allergic airway inflammation [42]. In both murine and human systems, HA drives regular T-cell precursors toward Foxp-IL-10-producing regulatory T cells (TR1), suggesting another mechanism for HA-upregulated IL-10 in vivo [43]. Conversely, IL-10 may regulate HA production in certain cases. For example, IL-10 mediates HA-rich ECM deposition in murine fetal fibroblasts [44]. Besides IL-10, the upregulation of SOCS1 was noticed after HMW-HA and PM_2.5_ co-treatment (Figure S1). SOCS1 negatively regulates inflammatory cytokine signaling by blocking JNK, p38, and NF-κB [45]. Further experiments may be conducted to verify the overproduction of SOCS1 upon HMW-HA treatment and investigate the downstream target of SOCS1.

The effect of HA on macrophages is dependent on the molecular sizes. Rayahin et al. demonstrated that, in general, LMW-HA drives macrophages toward M1 polarization, while HMW-HA exhibits an opposite property [25]. In our study, macrophages co-administered with HMW-HA and PM_2.5_ only expressed a high amount of IL-10, not MRC1 or Arg-1. Although IL-10 is a biomarker of M2 polarization, it is not specific enough to identify M2 macrophages as M1 macrophages also secrete IL-10. It is possible that PM_2.5_ has an inhibitory effect on HMW-HA-driven M2 polarization, and therefore, we were unable to observe a notable increase in M2 marker expression. In addition, we observed a minor increase in the expression of TNFA and IL10 when macrophages were treated with HMW-HA alone. According to previous research, endotoxin-free pharmaceutical grade HMW-HA fails to stimulate macrophages to secrete TNF-α [46]. The modest change in cytokine expression caused by HMW-HA in our experiment is probably due to the preparation of HA solution. Further study should be conducted in animals to evaluate the safety of HMW-HA systemically.

The most studied HA-interacting receptor is CD44. The HA-CD44 cross-linking functions as an anti-inflammatory signal promoting the production of cytokines related to resolution of inflammation and negatively regulating TLR signaling [23]. Modified HMW-HA also interacts with CD44 to inhibit NF-κB activation, dendritic cell maturation, and allergen-specific CD4 T-helper reaction, potentially providing therapeutic effects to asthma and allergic sinusitis [42]. In endothelial cells, HA mediates caveolin-enriched microdomains (CEM) via CD44 to decrease vascular permeability [20]. In differentiated THP-1 macrophages stimulated with TLR2, HA treatments reduced NF-κB nuclear translocation, and production of IL-1β and TNF-α by CD44 [47]. In addition, HMW-HA mitigates oxidative damage during inflammation. The other HA binding proteins may also contribute to the anti-inflammatory property of HMW-HA. Our next step is to investigate the function of various HA-binding proteins in HMW-HA-mediated anti-inflammatory action, and determine the downstream signaling pathway.

## 4. Materials and Methods

### 4.1. PM_2.5_ Collection and Preparation

Fine particulate matter was collected and prepared as described in our previous study [25]. Briefly, PM_2.5_ samples were collected with PM_2.5_ high volume air sampler (Thermon Anderson, Waltham, MA, USA) using quartz fiber filters (General Electric, Boston, MA, USA) at a flow rate of 1.13 m^3^/min for 24 h per day. After sampling, filters were cut and immersed in sterile distilled water, and then administered by ultrasonic sonication for four cycles of 20 min each. Particle suspensions were vacuum-freeze dried, weighed, and stored at −20 °C. PM_2.5_ was resuspended in sterile normal saline (NS) or phosphate buffer saline (PBS) (10 mg/mL), and was always sonicated and vortexed prior to use.

### 4.2. Cell Culture 

**Figure 7:**
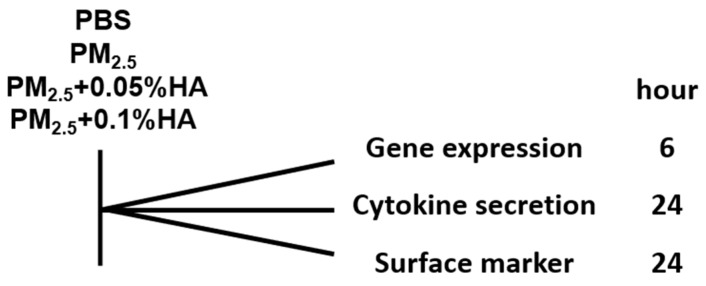
Timeline of in vitro study.

NR8383 rat alveolar macrophage was purchased from the Type Culture Collection (Chinese Academy of Sciences, Shanghai, China). Cells were maintained in Ham’s F-12K medium supplemented with 15% FBS (Gibco, Thermo Fisher Scientific, Inc., Waltham, MA, USA) and 1% penicillin/streptomycin (Thermo Fisher Scientific, Inc., Waltham, MA, USA) at 37 °C in a humidified atmosphere with 5% of CO_2_. Cells were treated with PBS, PM_2.5_, PM_2.5_ + 0.05%HA or PM_2.5_ + 0.1%HA for indicated hours for further assays (Figure 7).

### 4.3. RNA Isolation and Quantitative Real-time RT-PCR

Total RNA was extracted using GeneJET RNA Purification kit (Thermo Fisher Scientific, Inc., Waltham, MA, USA), according to manufacturer’s instruction, and then reverse-transcribed in 20 μL volume using RevertAid First Strand cDNA Synthesis kit (Thermo Fisher Scientific, Inc., Waltham, MA, USA). Real-time PCR was carried out using Applied Biosystems^®^ Real-time PCR system (Thermo Fisher Scientific, Inc., Waltham, MA, USA) with BeyoFast™ SYBR Green qPCR Mix (2X, Low ROX) (Beyotime Biotechnology, Nantong, China), forward and reverse primers in a final PCR reaction volume of 20 μL. Amplification was performed according to the manufacturer’s recommendation. The primers used in the present study are listed in Table 1.

### 4.4. Western Blot Analysis

Cells were lysed in RIPA buffer supplemented with 1 mM of PMSF (Thermo Fisher Scientific, Inc., Waltham, MA, USA). The protein extracts were loaded, separated by SDS polyacrylamide gel electrophoresis, and transferred to a polyvinylidene fluoride (PVDF) membrane (Bio-Rad Laboratories, Hercules, CA, USA). Membranes were blocked with 5% fat free milk in PBST (Beijing Solarbio Sciences & Technology Co., Ltd., Beijing, China) and incubated with primary antibodies against p-p38, p38, p-JNK, and JNK (Abcam, Cambridge, MA, USA) overnight at 4 °C. Protein bands were detected by incubation with horseradish peroxidase-conjugated antibodies and visualized with Clarity™ Western ECL Substrate (Bio-Rad Laboratories, Hercules, CA, USA).

### 4.5. Flow Cytometry

Cells were treated with PBS, PM_2.5_, or PM_2.5_ + 0.1%HA for 24 h and harvested by scrapping. Approximately 1 × 10^6^ cells were transferred to 1.5 mL tube, washed with PBS containing 10% FBS and 1% sodium azide (NaN_3_) twice, and incubated with 10 μg/mL CD86-FITC antibody (Abcam, Cambridge, MA, USA) in 3% BSA/PBS at room temperature in the dark for 30 min. Then cells were washed three times with PBS, 10% FBS, 1% NaN_3_ by centrifugation and resuspended in 500 μL PBS, 10% FBS, and 1% NaN_3_. The FITC intensity of each sample was analyzed by Beckman CytoFLEX S (Beckman Coulter, Brea, CA, USA).

### 4.6. Collection of BALF and Lung Tissues from Animal Model

**Figure 8:**
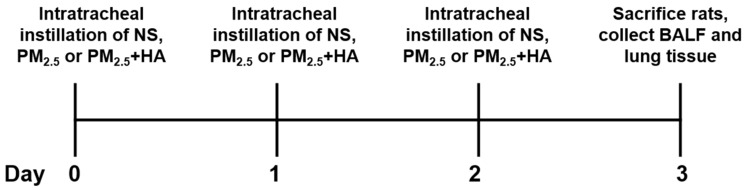
Timeline of animal experiments.

Eighteen male Sprague Dawley (SD) rats (200 ± 20 g) were obtained from Zhejiang Academy of Medical Sciences. The animals were housed in a specific pathogen-free (SPF) environment and maintained on standard diet with water ad libitum. After one week adaptation, the rats were weighed and randomly divided into three groups (N = 6): rats intratracheal instilled with normal saline (NS); rats received PM_2.5_ (8 mg/kg b.w.) and NS by intratracheal instillation; rats exposed to PM_2.5_ (8 mg/kg b.w.) and 200 μL of 0.2% HMW-HA (1500 kDa–1700 kDa; Sigma-Aldrich, St. Louis, MO, USA) by intratracheal instillation. Each group was treated as mentioned above for three consecutive days. Rats were sacrificed 24 h after last intratracheal instillation, and BALF and lung tissues were collected (Figure 8). All the animal experiments in this study were conducted with the approval of animal experiment center of Zhejiang University of Technology (approval no. 20171129031), and handled in accordance with the “Guide for the Care and Use of Laboratory Animals” and the “Principles for the Utilization and Care of Vertebrate Animals”.

### 4.7. Immunofluoresecence Staining

After dissection, rat lung tissues were fixed with 4% formalin and embedded in paraffin. Thin tissue sections (4 μm) were cut and the sections were placed on a glass slide. After deparaffinization, rehydration, and antigen retrieval, immunofluorescence was done according to manufacturer’s protocol. Anti-CD68 and anti-NOS2 antibodies (Proteintech Group, Chicago, IL, USA) were used as primary antibody. Alexa Fluor 594 (red) and Alexa Fluor 488 (green) conjugated secondary antibodies (Proteintech Group, Chicago, IL, USA) at a 1:200 (*v/v*) dilution were used. DAPI (4,6-diamidino-2-phenylindole) was used to stain nuclei. The slides were observed under an Olympus (USA) inverted fluorescent microscope.

### 4.8. ELISA Assay

The cytokine levels in the cell culture medium and the BALF were measured by commercially available ELISA assay kits (Neobioscience Technology Co., Ltd., Hong Kong, China). All experiments were performed according to the manufacturer’s protocol.

### 4.9. Statistical Method

Values are presented as mean ± SD or mean ± SEM. Data were compared through *t*-test or one-way analysis of variance (ANOVA). *p* < 0.05 was considered to be statistically significant.

## Figures and Tables

**Table 1:** Primers used in the present study

Genes	Primer Sequence (5′→3′)
TNFA	Forward primer: CCCAGACCCTCACACTCAGAT Reverse primer: TTGTCCCTTGAAGAGAACCTG
IL1B	Forward primer: ATCATCCCACGAGTCACAGAGG Reverse primer: TACCTATGTCTTGCCCGTGGAG
IL6	Forward primer: ACTTCACAGAGGATACCAC Reverse primer: GCATCATCGCTGTTCATAC
CXCL1	Forward primer: GCAGACAGTGGCAGGGATT Reverse primer: GGACACCCTTTAGCATCTTTT
CXCL2	Forward primer: TGCTCAAGACTCCAACCACTC Reverse primer: ACAACAACCCCTGTACCCTG
NOS2	Forward primer: CTATTCCCAGCCCAACAACA Reverse primer: CTGGAACATTCTGTGCAGTC
IL10	Forward primer: GAAAAATTGAACCACCCGGCA Reverse primer: TTCCAAGGAGTTGCTCCCGT
MRC1	Forward primer: CAAGGAAGGTTGGCATTTGT Reverse primer: GGAACGTGTGCTCTGAGTT
ARG1	Forward primer: ACAAGACAGGGCTACTTTCAGG Reverse primer: ACAAGACAAGGTCAACGCCA
SOCS1	Forward primer: CCTTCGACTGCCTCTTCGAG Reverse primer: AGTCACGGAGTACCGGGTTA
Endogenous control (ACTB)	Forward primer: AGCCATGTACGTAGCCATCC Reverse primer: ACCCTCATAGATGGGCACAG

## Supplementary Materials

The following are available online, Figure S1: HMW-HA up-regulates SOCS1 mRNA level in PM2.5-stimulated NR8383 cells.

## References

[1] Feng S., Gao D., Liao F., Zhou F., Wang X. (2016). The health effects of ambient PM2.5 and potential mechanisms. Ecotoxicol. Environ. Saf..

[2] Falcon-Rodriguez C.I., Osornio-Vargas A.R., Sada-Ovalle I., Segura-Medina P. (2016). Aeroparticles, Composition, and Lung Diseases. Front. Immunol..

[3] Ma Q.Y., Huang D.Y., Zhang H.J., Wang S., Chen X.F. (2017). Exposure to particulate matter 2.5 (PM2.5) induced macrophage-dependent inflammation, characterized by increased Th1/Th17 cytokine secretion and cytotoxicity. Int. Immunopharmacol..

[4] Ni L., Chuang C.C., Zuo L. (2015). Fine particulate matter in acute exacerbation of COPD. Front. Physiol..

[5] Zhao Q., Chen H., Yang T., Rui W., Liu F., Zhang F., Zhao Y., Ding W. (2016). Direct effects of airborne PM2.5 exposure on macrophage polarizations. Biochim. Biophys. Acta.

[6] Wang N., Liang H., Zen K. (2014). Molecular mechanisms that influence the macrophage m1-m2 polarization balance. Front. Immunol..

[7] Murray P.J. (2017). Macrophage Polarization. Annu. Rev. Physiol..

[8] Chistiakov D.A., Myasoedova V.A., Revin V.V., Orekhov A.N., Bobryshev Y.V. (2018). The impact of interferon-regulatory factors to macrophage differentiation and polarization into M1 and M2. Immunobiology.

[9] Sica A., Erreni M., Allavena P., Porta C. (2015). Macrophage polarization in pathology. Cell. Mol. Life Sci..

[10] Saradna A., Do D.C., Kumar S., Fu Q.L., Gao P. (2018). Macrophage polarization and allergic asthma. Transl. Res..

[11] He M., Ichinose T., Yoshida Y., Arashidani K., Yoshida S., Takano H., Sun G., Shibamoto T. (2017). Urban PM2.5 exacerbates allergic inflammation in the murine lung via a TLR2/TLR4/MyD88-signaling pathway. Sci. Rep..

[12] Shoenfelt J., Mitkus R.J., Zeisler R., Spatz R.O., Powell J., Fenton M.J., Squibb K.A., Medvedev A.E. (2009). Involvement of TLR2 and TLR4 in inflammatory immune responses induced by fine and coarse ambient air particulate matter. J. Leukoc. Biol..

[13] Tan H.Y., Wang N., Li S., Hong M., Wang X., Feng Y. (2016). The Reactive Oxygen Species in Macrophage Polarization: Reflecting Its Dual Role in Progression and Treatment of Human Diseases. Oxid. Med. Cell Longev..

[14] Wu X., Gao H., Hou Y., Yu J., Sun W., Wang Y., Chen X., Feng Y., Xu Q.M. (2018). Dihydronortanshinone, a natural product, alleviates LPS-induced inflammatory response through NF-kappaB, mitochondrial ROS, and MAPK pathways. Toxicol. Appl. Pharmacol..

[15] Cowman M.K. (2017). Hyaluronan and Hyaluronan Fragments. Adv. Carbohydr. Chem. Biochem..

[16] Tavianatou A.G., Caon I., Franchi M., Piperigkou Z., Galesso D., Karamanos N.K. (2019). Hyaluronan: molecular size-dependent signaling and biological functions in inflammation and cancer. FEBS J..

[17] Litwiniuk M., Krejner A., Speyrer M.S., Gauto A.R., Grzela T. (2016). Hyaluronic Acid in Inflammation and Tissue Regeneration. Wounds.

[18] Hussain S., Ji Z., Taylor A.J., DeGraff L.M., George M., Tucker C.J., Chang C.H., Li R., Bonner J.C., Garantziotis S. (2016). Multiwalled Carbon Nanotube Functionalization with High Molecular Weight Hyaluronan Significantly Reduces Pulmonary Injury. ACS Nano.

[19] Lu K.W., Goerke J., Clements J.A., Taeusch H.W. (2005). Hyaluronan reduces surfactant inhibition and improves rat lung function after meconium injury. Pediatr. Res..

[20] Singleton P.A., Mirzapoiazova T., Guo Y., Sammani S., Mambetsariev N., Lennon F.E., Moreno-Vinasco L., Garcia J.G. (2010). High-molecular-weight hyaluronan is a novel inhibitor of pulmonary vascular leakiness. Am. J. Physiol. Lung Cell. Mol. Physiol..

[21] He M., Ichinose T., Kobayashi M., Arashidani K., Yoshida S., Nishikawa M., Takano H., Sun G., Shibamoto T. (2016). Differences in allergic inflammatory responses between urban PM2.5 and fine particle derived from desert-dust in murine lungs. Toxicol. Appl. Pharmacol..

[22] Johnson P., Arif A.A., Lee-Sayer S.S.M., Dong Y. (2018). Hyaluronan and Its Interactions With Immune Cells in the Healthy and Inflamed Lung. Front. Immunol..

[23] Ruppert S.M., Hawn T.R., Arrigoni A., Wight T.N., Bollyky P.L. (2014). Tissue integrity signals communicated by high-molecular weight hyaluronan and the resolution of inflammation. Immunol. Res..

[24] Xu C., Shi Q., Zhang L., Zhao H. (2018). High molecular weight hyaluronan attenuates fine particulate matter-induced acute lung injury through inhibition of ROS-ASK1-p38/JNK-mediated epithelial apoptosis. Environ. Toxicol. Pharmacol..

[25] Rayahin J.E., Buhrman J.S., Zhang Y., Koh T.J., Gemeinhart R.A. (2015). High and low molecular weight hyaluronic acid differentially influence macrophage activation. ACS Biomater. Sci. Eng..

[26] Liu C.P., Zhang X., Tan Q.L., Xu W.X., Zhou C.Y., Luo M., Li X., Huang R.Y., Zeng X. (2017). NF-kappaB pathways are involved in M1 polarization of RAW 264.7 macrophage by polyporus polysaccharide in the tumor microenvironment. PLoS ONE.

[27] Tian L., Li W., Yang L., Chang N., Fan X., Ji X., Xie J., Li L. (2017). Cannabinoid Receptor 1 Participates in Liver Inflammation by Promoting M1 Macrophage Polarization via RhoA/NF-kappaB p65 and ERK1/2 Pathways, Respectively, in Mouse Liver Fibrogenesis. Front. Immunol..

[28] Kou X.X., Li C.S., He D.Q., Wang X.D., Hao T., Meng Z., Zhou Y.H., Gan Y.H. (2015). Estradiol promotes M1-like macrophage activation through cadherin-11 to aggravate temporomandibular joint inflammation in rats. J. Immunol..

[29] Zhong J., Wang H., Chen W., Sun Z., Chen J., Xu Y., Weng M., Shi Q., Ma D., Miao C. (2017). Ubiquitylation of MFHAS1 by the ubiquitin ligase praja2 promotes M1 macrophage polarization by activating JNK and p38 pathways. Cell Death Dis..

[30] He M., Ichinose T., Yoshida S., Ito T., He C., Yoshida Y., Arashidani K., Takano H., Sun G., Shibamoto T. (2017). PM2.5-induced lung inflammation in mice: Differences of inflammatory response in macrophages and type II alveolar cells. J. Appl. Toxicol..

[31] Zhang J., Zeng X., Li Y., Zhao W., Chen Z., Du Q., Zhou F., Ji N., Huang M. (2019). Exposure to Ambient Particles Alters the Evolution of Macrophage Phenotype and Amplifies the Inducible Release of Eotaxin-1 in Allergen-Sensitized Mice. J. Biomed. Nanotechnol..

[32] Zhu X., Zhao P., Lu Y., Huo L., Bai M., Yu F., Tie Y. (2019). Potential injurious effects of the fine particulate PM2.5 on the progression of atherosclerosis in apoE-deficient mice by activating platelets and leukocytes. Arch. Med. Sci..

[33] Krocova Z., Macela A., Kroca M., Hernychova L. (2000). The immunomodulatory effect(s) of lead and cadmium on the cells of immune system in vitro. Toxicol. In Vitro.

[34] Riemschneider S., Kohlschmidt J., Fueldner C., Esser C., Hauschildt S., Lehmann J. (2018). Aryl hydrocarbon receptor activation by benzo(a)pyrene inhibits proliferation of myeloid precursor cells and alters the differentiation state as well as the functional phenotype of murine bone marrow-derived macrophages. Toxicol. Lett..

[35] Hansen J.F., Nielsen C.H., Brorson M.M., Frederiksen H., Hartoft-Nielsen M.L., Rasmussen A.K., Bendtzen K., Feldt-Rasmussen U. (2015). Influence of phthalates on in vitro innate and adaptive immune responses. PLoS ONE.

[36] Rendra E., Riabov V., Mossel D.M., Sevastyanova T., Harmsen M.C., Kzhyshkowska J. (2018). Reactive oxygen species (ROS) in macrophage activation and function in diabetes. Immunobiology.

[37] Quinn S.R., O’Neill L.A. (2014). The role of microRNAs in the control and mechanism of action of IL-10. Curr. Top. Microbiol. Immunol..

[38] Tarantino G., Caputi A. (2011). JNKs, insulin resistance and inflammation: A possible link between NAFLD and coronary artery disease. World J. Gastroenterol..

[39] Yang Y., Kim S.C., Yu T., Yi Y.S., Rhee M.H., Sung G.H., Yoo B.C., Cho J.Y. (2014). Functional roles of p38 mitogen-activated protein kinase in macrophage-mediated inflammatory responses. Mediat. Inflamm..

[40] Saraiva M., O’Garra A. (2010). The regulation of IL-10 production by immune cells. Nat. Rev. Immunol..

[41] Burmeister A.R., Marriott I. (2018). The Interleukin-10 Family of Cytokines and Their Role in the CNS. Front. Cell. Neurosci..

[42] Gebe J.A., Yadava K., Ruppert S.M., Marshall P., Hill P., Falk B.A., Sweere J.M., Han H., Kaber G., Harten I.A. (2017). Modified High-Molecular-Weight Hyaluronan Promotes Allergen-Specific Immune Tolerance. Am. J. Respir. Cell. Mol. Biol..

[43] Bollyky P.L., Wu R.P., Falk B.A., Lord J.D., Long S.A., Preisinger A., Teng B., Holt G.E., Standifer N.E., Braun K.R. (2011). ECM components guide IL-10 producing regulatory T-cell (TR1) induction from effector memory T-cell precursors. Proc. Natl. Acad. Sci. USA.

[44] King A., Balaji S., Le L.D., Marsh E., Crombleholme T.M., Keswani S.G. (2013). Interleukin-10 regulates fetal extracellular matrix hyaluronan production. J. Pediatr. Surg..

[45] Yoshimura A., Ito M., Chikuma S., Akanuma T., Nakatsukasa H. (2018). Negative Regulation of Cytokine Signaling in Immunity. Cold Spring Harb. Perspect. Biol..

[46] Dong Y., Arif A., Olsson M., Cali V., Hardman B., Dosanjh M., Lauer M., Midura R.J., Hascall V.C., Brown K.L. (2016). Endotoxin free hyaluronan and hyaluronan fragments do not stimulate TNF-alpha, interleukin-12 or upregulate co-stimulatory molecules in dendritic cells or macrophages. Sci. Rep..

[47] Qadri M., Almadani S., Jay G.D., Elsaid K.A. (2018). Role of CD44 in Regulating TLR2 Activation of Human Macrophages and Downstream Expression of Proinflammatory Cytokines. J. Immunol..

