# Supplementary material for: High Molecular Weight Hyaluronan Suppresses Macrophage M1 Polarization and Enhances IL-10 Production in PM2.5-Induced Lung Inflammation
Source: Molecules. 2019 May 7;24(9):1766. doi: 10.3390/molecules24091766 (PMC6539614; doi:10.3390/molecules24091766)
Supplement: Supplementary file 1 [file molecules-24-01766-s001.pdf]

Supplementary Materials:

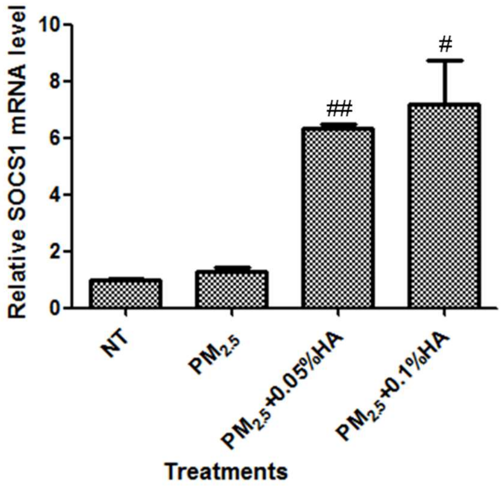

Figure S1 HMW-HA up-regulates SOCS1 mRNA level in $PM_{2.5}$-stimulated NR8383 cells. Cells were exposed to PBS, $PM_{2.5}$, $PM_{2.5}$ and 0.05% HMW-HA, or $PM_{2.5}$ and 0.1% HMW-HA simultaneously for 6 h, and the mRNA expression of SOCS1 was determined by real-time RT-PCR. Data are presented as mean ± SD, and represent three independent experiments. # $p < 0.05$ and ## $p < 0.01$, compared with cells exposed to $PM_{2.5}$ alone.
